# Recurrent retroperitoneal extra-renal angiomyolipoma: A case report and review of literature

**DOI:** 10.1016/j.amsu.2022.104230

**Published:** 2022-07-31

**Authors:** Narjes Mohammadzadeh, Soroush Kohansal, Amir ghasemlouei

**Affiliations:** a Department of Surgery, Imam Khomeini Hospital, Tehran University of Medical Sciences, Tehran, Iran; b Shahed University, Tehran, Iran

**Keywords:** Angiomyolipoma, Extra-renal angiomyolipoma

## Abstract

**Introduction:**

Angiomyolipoma (AML) is the most prevalent renal mesenchymal neoplasm that almost always involves kidneys.

**Case presentation:**

We present a rare condition of retroperitoneal extra-renal angiomyolipoma which relapsed after 4 years that caused acute abdominal pain.

**Discussion:**

The liver is the most common site of extrarenal angiomyolipoma. Abdominal pain is the most common complaint of extra-renal angiomyolipoma. Computed tomography angiography is the best way for angiomyolipoma diagnosis and surgery and post-surgery chemotherapy is the way for its treatment.

**Conclusion:**

extrarenal angiomyolipoma is an extremely rare condition that patients should be followed up for over 5 years by appropriate radiologic imaging.

## Highlights

1).
  • Angiomyolipoma almost always involves kidneys.
  • We present a retroperitoneal extra-renal angiomyolipoma case.
2)
  • extra-renal angiomyolipoma is characterized as a benign tumor.
  • We present a recurrent retroperitoneal extra-renal angiomyolipoma.
3) surgery is one of the best ways to both diagnosis and treatment of retroperitoneal extra-renal angiomyolipoma.

## 1 Introduction

Angiomyolipoma (AML) is the most prevalent renal mesenchymal neoplasm that is described by perivascular epithelioid cells that co-express smooth muscle and melanogenesis markers [1]. Although AML almost always involves kidneys few articles indicate the possibility of extra-renal involvement as an extremely rare entity [2]. Histologic findings in both renal and extra-renal AML (ERAML) are similar but the renal type is more aggressive and may relapse [3].

In this case report, we present a rare condition of retroperitoneal ERAML which relapsed after 4 years that caused acute abdominal pain and bleeding in a 43-year-old woman.

## 2 Case presentation

A 43-year-old female, a known case of retroperitoneal AML, was referred to the hospital with a complaint of abdominal pain for 5 days. The pain was initially in the periumbilical area and then accumulated in the right lower quadrant and referred to the left lower quadrant and back. It was reduced by bending forward and eating and had increased on the last day. She did not have any symptoms of nausea, vomiting, diarrhea, chest pain, and dyspnea. She did not mention any specific drug, family, or social history. Her past medical history was prominent for retroperitoneal AML next to the right renal arteries, 4 years ago, which was surgically removed. Radiotherapy was offered to him but she was refused this treatment.

Upon arrival, she was not ill or toxic. Her vital signs were blood pressure:110/90 mmHg, pulse rate:85/min, temperature:36.7°, respiratory rate: 17/min, and O2 saturation:95%. In the abdominal examination, she had mild generalized tenderness without rebound, guarding, organomegaly, or distention. Her laboratory tests are shown in Table 1. Computed tomography angiography (CTA) was requested for her which showed heterogenic enhancing mass with cystic or necrotic changes in the anterior side of the abdominal aorta from the uncinate process of the pancreas to the iliac bifurcation.

**Table 1:** Laboratory tests.

test	result	unit	normal	test	result	unit	normal
RBC	3.52	M/mm^3^	4.2–5.8	Na	140	meq/L	135–145
Hct	30.9	%	36–51	K	3.9	meq/L	3.5–5.5
HB	10.3	g/dL	12–16	UREA	29	mg/dL	15–50
WBC	6.4	10^3^/mm^3^	4.1–10.1	BS	101	mg/dL	<200
PLT	187	10^3^/mm^3^	150–400	CRP	5	mg/L	<6.0
PT	13	sec	11–15	ALP	161′	U/L	70–360
PTT	27	sec	25–45	ALT	16	U/L	<31
Amylase	55	U/L	<100	AST	15	U/L	<31
Lipase	40	U/L	<60	Bill.T	1.7	mg/dL	0.1–1.2
troponin-hs	<0.2	ng/ml	<0.29	Bill.D	0.6	mg/dL	<0.3

**Fig. 1:**
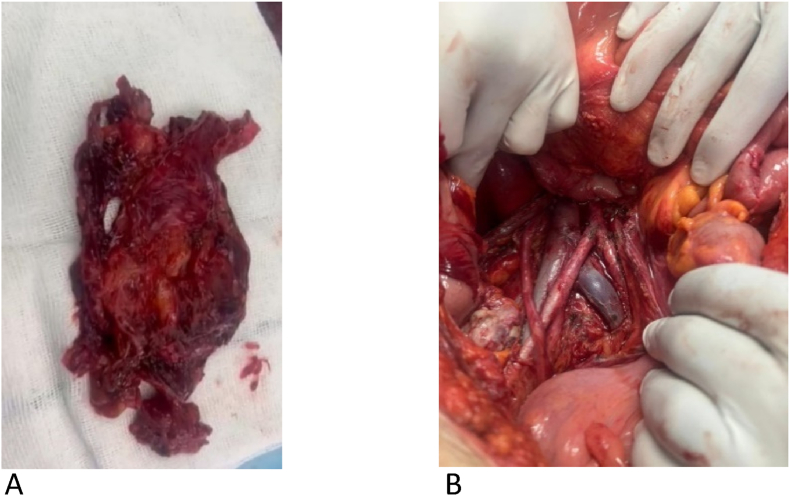
A: angiomyolipoma after removing from Retroperitoneal viscera. B. IVC and aorta bifurcation exposed after removing the mass.

According to the current situation, previous history, and radiological findings of the patient, a diagnostic laparotomy was performed which showed 500 ccs of bloody fluid in the peritoneal and pelvic cavity. Medial rotation of right colon done. Retroperitoneal fossa explored and an extended mass was found which was lying on the back of the abdomen. The mass was isolated and separated from the aorta and inferior vena cava. The upper edge of the tumor was limited to the pancreas and duodenum with an appropriate plane between them. The tumor was removed completely (Fig. 1) and was sent to the pathology laboratory (Table 2).

**Table 2:** Pathologic report.

Block summary	Pathologic diagnosis
“Retroperitoneal tumor”, consists of multiple pieces of fibrofatty tan tissue measuring 11 × 5 × 3 cm.	- Angiomyolipoma, epithelioid type, recurrence - Tumor size:11 × 5 × 3 cm - Mitosis count:0–1/10 HPF - Extensive areas of hemorrhage were also noted - Two reactive lymph nodes, free from tumor

## 3 Discussion

AMLs are benign tumors that have the triad of abnormal blood vessels, muscle fibers, and mature adipose tissue [4]. They belong to the family of perivascular epithelioid cell tumors (PEComas). A tumor of this family is distinguished by the unique morphology of perivascular epithelioid cells (PECs) and a “myomelanocytic immunophenotype” [5].

Minija EJ. considered 56 cases of ERAML (all reported cases from 1982 to 2011). They found 18 cases of liver AML (34.61%), 16 cases of retroperitoneum AML (30.76%), 7 cases of uterus AML (13.46%), 2 cases of vagina AML (3.84%), and 2 cases of head involvement (3.84%). Each Hard plate, abdominal wall, penis, fallopian tube, nasal cavity, spermatic cord, and colon AML was reported as a unique case [6].

Venyo AK. considered 30 cases of retroperitoneal ERAML in 2016 (all known cases up to 2016). According to the site of involvement, there were different clinical symptoms such as abdominal pain (most compliant (14 cases) – similar to our case), bleeding, masses, back pain, weight loss, abdominal distension, weight gain, vomiting, constipation, hematuria, and urinary frequency. Also, there were three cases of accidental findings [7].

Laboratory investigations (hematological tests, serum biochemistry tests, urine analysis, urine microscopy, and urine culture) are basic tests undertaken in the assessment of this patient but because of nonspecific findings, they are not helpful for ERAML diagnosis [7].

CTA is the best radiologic method for ERAML diagnosis [6]. Retroperitoneal ERAMLs typically indicate aneurysmal dilatation of the intramural vessels, intramural linear vascularity, bridging veins beak sign, hematomas, and discrete intrarenal or extrarenal fatty tumors, but none of these are pathogenic [8]. Abdominal & pelvic sonography can be helpful for ERAML diagnosis. It may show the mass in the retroperitoneum, indicate the size and extent of the tumor, and whether or not the tumor mass has displaced any nearby organs [7]. MRI can be used in addition to CTA which shows the anatomical features [6].

Surgery is the common procedure for ERAML treatment [7]. If the tumor is near the kidneys, it is necessary to engage a histological frozen section to defend the renal system [9]. In patients whose retroperitoneal bleeding causes an emergency, selective arterial angiography and embolization have been undertaken effectively to control bleeding which tends to allow for elective surgical excision [7].

## 4 Conclusion

Although ERAML is characterized as a benign tumor, it may involve liver or lymph nodes as metastatic features. Therefore, it may be good for patients to be followed up over 5 years by appropriate radiological imaging to minimize the risk of recurrence involvement [7].

## Sources of funding

There is no financial support statement.

## Ethical approval

Nothing to declare.

## Consent

Written informed consent was obtained from the patient for publication of this case report and accompanying images. A copy of the written consent is available for review by the Editor-in-Chief of this journal on request.

## Author contribution

Narjes Mohammadzadeh: Chief surgeon, concept and design, final revision.

Soroush Kohansal: writing the paper.

Amir ghasemlouei: Surgeon, final revision.

## Registration of research studies

Name of the registry: N/A.
Unique Identifying number or registration ID: N/A.
Hyperlink to your specific registration (must be publicly accessible and will be checked): N/A.

## Guarantor

Amir Ghasemlouei.

## Declaration of competing interest

All authors declare that there is no conflict of interest regarding the publication of this paper.
